# Tricuspidization technique with reimplantation for a bicuspid aortic valve: a case report

**DOI:** 10.1186/s44215-022-00005-2

**Published:** 2022-09-27

**Authors:** Yu Hohri, Satoshi Numata, Yutaka Okita, Hitoshi Yaku

**Affiliations:** 1 Department of Cardiovascular Surgery, Kyoto Prefectural University of Medicine, 465 Kajii-cho, Kawaramachi Hirokoji, Kamigyo-ku, Kyoto, 602-8566 Japan; 2 Cardio-Aortic Centre, Takatsuki General Hospital, Osaka, Japan

**Keywords:** Bicuspid aortic valve, Aortic regurgitation, Tricuspidization, Aortic valve repair, Valve-sparing aortic root replacement

## Abstract

**Background:**

Bicuspid aortic valve (BAV) is the most frequent congenital cardiac anomaly. We report a successful case in which the tricuspidization technique with valve-sparing aortic root replacement was used for BAV with severe aortic regurgitation.

**Case presentation:**

A 22-year-old man was admitted for progressively worsening aortic regurgitation due to a congenital BAV. Preoperative examination revealed annuloaortic ectasia and left ventricular dilatation with worsening ejection fraction. The right and left coronary cusps of the aortic valve were fused with severe prolapse. During surgery, as there was no obvious calcification or degeneration of each coronary cusp and the commissural orientation was nearly 120°, we judged that tricuspid reconstruction was preferable. Neo-left and right commissure reconstruction by raphe suspension and free margin resuspension of the non-coronary cusp were performed with valve-sparing aortic root replacement (reimplantation technique). Postoperatively, the coaptation height of each coronary cusp was remarkably increased, and aortic regurgitation and left ventricular function improved. The patient was discharged 12 days postoperatively without any complications.

**Conclusions:**

The tricuspidization technique with valve-sparing aortic root replacement was a valuable strategy for repairing the bicuspid valve (type I) with severe aortic valve regurgitation. Although we believe that our tricuspidization technique has the potential for good durability, further experience is warranted to confirm the safety and efficacy of this technique.

**Supplementary Information:**

The online version contains supplementary material available at 10.1186/s44215-022-00005-2.

## Background

Aortic valve repair (AVP) has remarkable benefits in young patients with severe aortic regurgitation (AR) to avoid life-long anticoagulation or complications of prosthetic valves. We report a successful case in which the tricuspidization technique with valve-sparing aortic root replacement (reimplantation technique) was used to treat severe AR due to a congenital bicuspid aortic valve (BAV).

## Case presentation

**Fig. 1:**
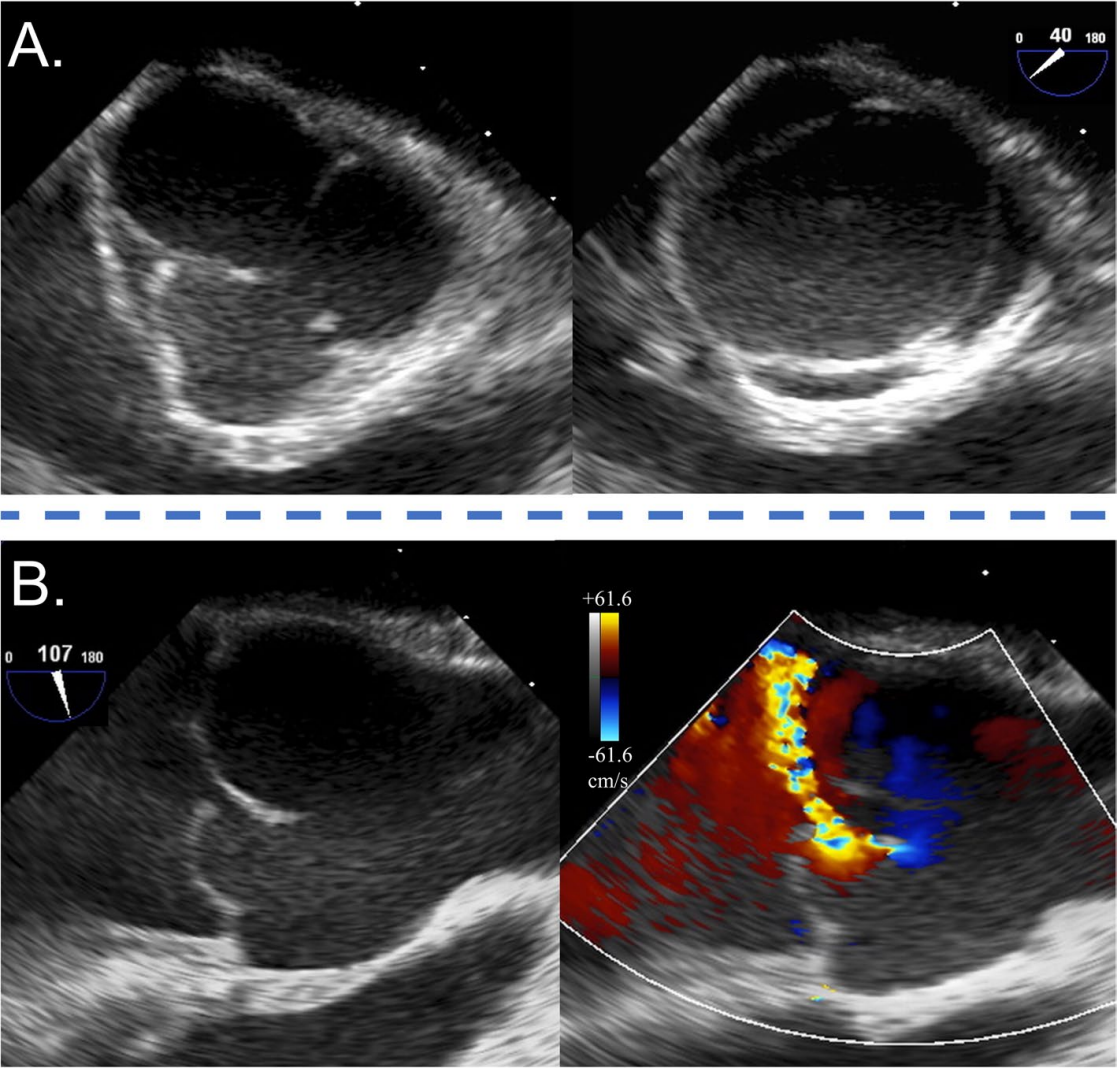
Preoperative transesophageal echocardiography. **A** Short-axis imaging in diastolic and systolic phases. **B** Long-axis B-mode and color Doppler imaging during the diastolic phase

A 22-year-old male patient, who had been diagnosed with AR due to BAV at 11 years of age, was admitted to our hospital. Although he was asymptomatic, his last echocardiography had shown worsening left ventricular ejection fraction (LVEF) by 54% and left ventricular (LV) dilatation (LV end-diastolic/systolic diameter [LVDd/LVDs], 63.9 mm/45.6 mm). Furthermore, the left and right coronary cusps were fused (Fig. 1A), and the fused cusp was severely prolapsed (Fig. 1B). The eccentric aortic regurgitant jet was striking the anterior mitral leaflet and the vena contracta was 6.4 mm, leading to the diagnosis of severe AR (Fig. 1B). Computed tomography showed that the sinus of Valsalva was dilated to 41.0 mm, and the diameters of the ventriculoaortic and sinotubular junctions were 31.0 and 28.3 mm, respectively. Therefore, we concluded that the aortic root dilatation and prolapse of the fused cusp caused severe AR.

**Fig. 2:**
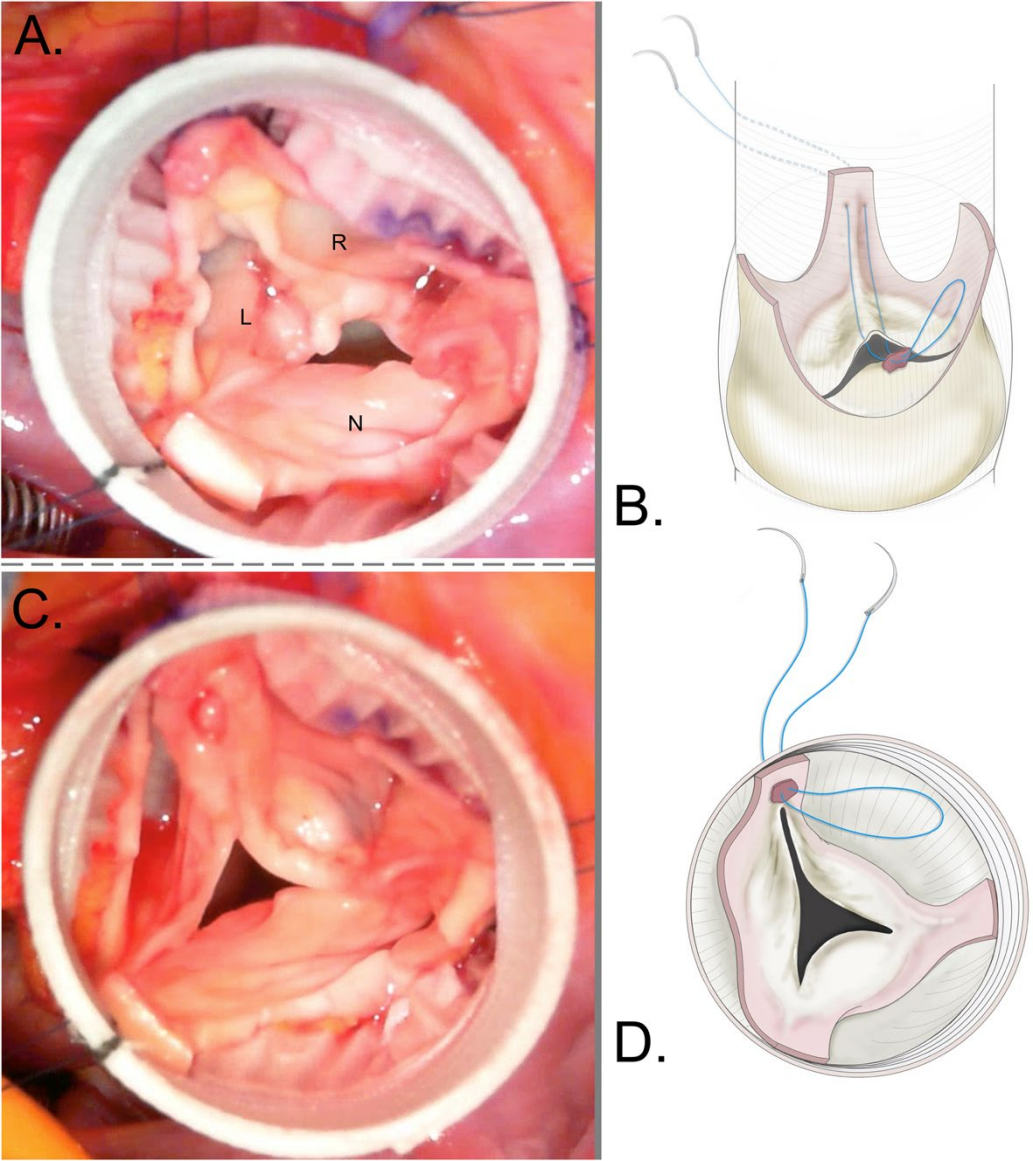
Intraoperative imaging and operative scheme of the tricuspidization technique. **A** Pre-aortic valve repair: the right and left coronary cusps are fused and severely prolapsed. L left coronary cusp, R right coronary cusp, N non-coronary cusp. **B** Scheme of the neo-commissure reconstruction. A horizontal mattress suture is placed at the free margin of the raphe. The ends of the sutures are passed outside the prosthetic graft at the level of the sinotubular junction. **C**, **D** Postoperative imaging of the neo-commissure reconstruction

The patient underwent AVP and valve-sparing aortic root replacement (reimplantation technique) via a median full sternotomy. After the aortic valve was exposed through a transverse aortotomy under cardiopulmonary bypass, the right and left coronary cusps were fused and severely prolapsed (geometric height: 20, 22, and 26 mm for the left, right, and non-coronary cusp, respectively). The diameter of the Brussel’s height was 24 mm, and the ventriculoaortic junction was 25 mm. Each coronary cusp was almost equal in size with a commissural orientation of nearly 120°, which is classified as the very asymmetrical bicuspid aortic valve with fusion of right and left coronary cusps [1, 2] (Fig. 2A). After applying the first row of sutures to a Gelweave Valsalva Ante-Flo Gelatin Impregnated Woven Dacron Graft 26-mm (Terumo Vascutek, Tokyo, Japan), a horizontal mattress suture using 5–0 Nespilene (Alfresa Holding Corporation, Tokyo, Japan) was placed at the free margin of the raphe. We decided the ideal height of the neo-commissure based on the height of other commissures, and the ends of the sutures were passed outside the prosthetic graft at the level of the sinotubular junction (neo-commissure reconstruction) (Fig. 2B). After neo-commissure reconstruction and placement of the second row of sutures, the original non-coronary cusp became prolapsed compared to the neo-left and right coronary cusps because the non-coronary cusp leaflet was larger than the other cusp leaflets (effective height: 8, 8, and 3 mm for the left, right, and non-coronary cusps, respectively) (Fig. 2C, D). Therefore, double-row continuous mattress sutures were applied in and out of the free margin of the non-coronary cusp leaflet with 7–0 Gore-Tex (W. L. Gore and Associates, Inc., Flagstaff, AZ) suture, and the tension of this suture was adjusted to equally correct the effective height of each cusp (free margin resuspension) (Additional file 1). Finally, the left and right coronary arteries were anastomosed to the prosthetic graft using the Carrel patch technique, and a distal anastomosis was performed between the prosthetic graft and the ascending aorta.

**Fig. 3:**
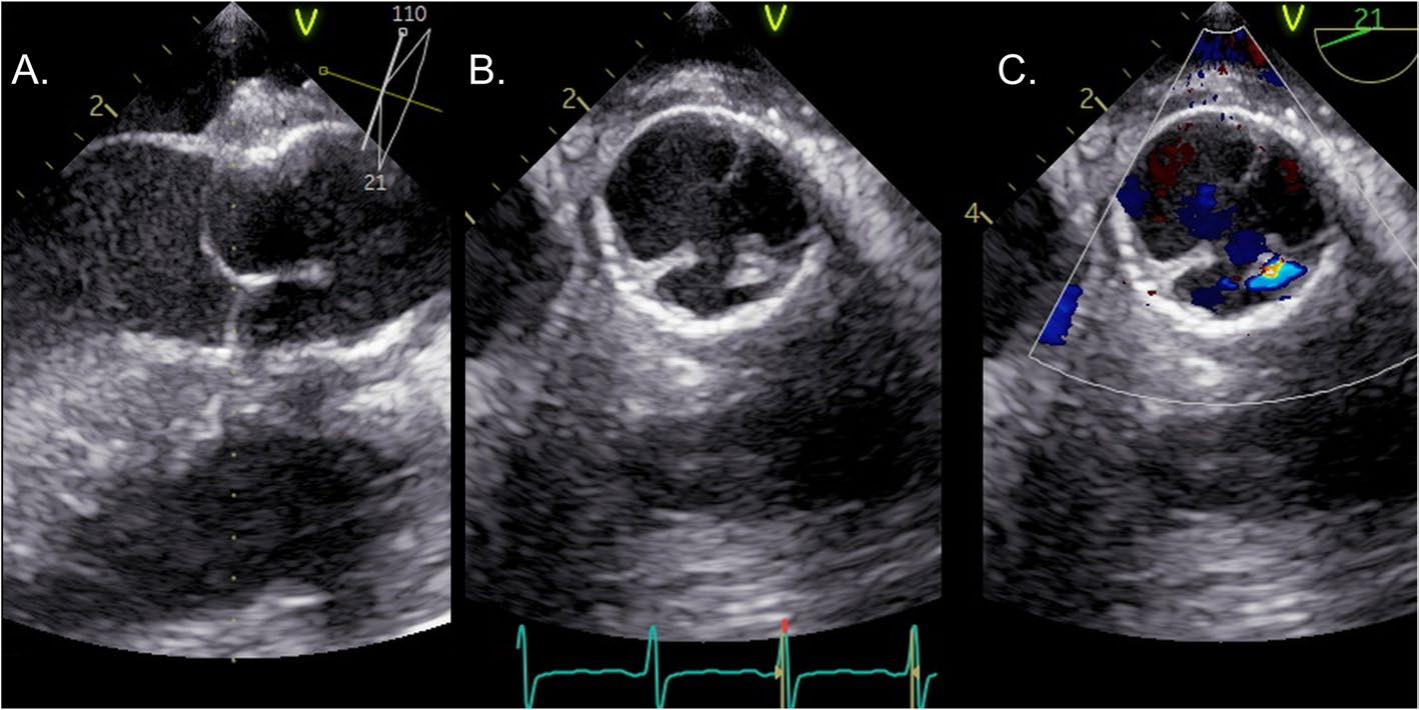
Postoperative transesophageal echocardiography. **A** Long-axis imaging during the diastolic phase. The coaptation of each coronary cusp appears remarkably improved. **B**, **C** Short-axis imaging during the diastolic phase. **B** B-mode imaging and **C** Color Doppler imaging. Mild regurgitation has occurred at the neo-commissure. The vena contracta is 1.0 mm

The patient’s postoperative course was uneventful. The echocardiography showed that LV dilatation had improved (LVDd: 54.0 mm; LVDs: 32.3 mm), and LVEF increased to 65.0%. Moreover, the effective height was sufficiently improved (8.7, 9.0, and 8.8 mm for the left, right, and non-coronary cusp, respectively). Mild regurgitation occurred at the neo-commissure, and the vena contracta of the AR jet was 1.0 mm, which was classified as mild AR (Fig. 3). The patient was discharged 12 days postoperatively without any complications. At present, 1.5 years after the surgery, the patient is well, and no progressive regurgitation or decreased left ventricular diameter was observed on the follow-up echocardiography.

## Discussion

This study describes our tricuspidization technique, a combination of raphe suspension and free margin resuspension, to repair a congenital BAV with severe AR. In our technique, the neo-commissure was reconstructed by suspending the raphe. The tricuspid reconstruction technique using the pericardium has been reported previously [3, 4]; however, the use of a pericardial patch has a negative effect on repair durability [5]. Our technique is simpler and only requires native valve tissue. Additionally, we performed free margin resuspension to improve the prolapse of the non-coronary cusp. Although foreign material can remain on the aortic cusp, postoperative valve restriction due to a fibrous reaction or calcification is extremely rare [6]; therefore, we believe that our technique has good durability.

Some studies advocate a repair strategy that repositions the commissures at a fully symmetrical orientation of 180° with valve-sparing aortic root replacement for BAV patients with aortic root dilatation [7, 8]. However, according to finite element analysis of the fluid-structure interaction, the BAV causes greater stress at the center of the valve leaflets than in the tricuspid aortic valve [9]. By making the valve tricuspid, the aortic valve may move more physiologically, its opening area may become larger, and the stress on the valve may be minimized [10]. Thus, for BAV with aortic root dilatation, we believe that tricuspidization repair can result in better hemodynamic improvement compared with bicuspidization repair.

Repair failure for BAV is influenced by the commissural orientation [5]. In cases of bicuspidization, reoperation was required significantly more often if commissural orientation was < 160° than if it were > 160° [5]. Furthermore, the aortic valve is best treated in analogy to the tricuspid aortic valve in patients with commissural orientation of < 140° [7]. With our technique, BAV with a raphe (Sievers classification; type I) is preferable, and the coronary cusps should be almost equal in size with no calcification or degeneration. Fortunately, in this case, there was no obvious calcification or degeneration of each coronary cusp, and the orientation was nearly 120°. Thus, the geometry of each coronary cusp is the key requirement for the successful performance of our technique. 

In conclusion, although the geometry of the coronary cusps was limited, our tricuspidization procedure with the reimplantation technique, which combined raphe suspension and free margin resuspension, can be an option for repairing a congenital BAV with aortic root dilatation. Further studies are warranted to confirm the safety and efficacy of this technique.

## Supplementary Information

**Additional file 1.** Tricuspidization technique with reimplantation for the congenital bicuspid aortic valve.

## Data Availability

The data underlying this article will be shared on reasonable request to the corresponding author.

## Abbreviations

AR: Aortic regurgitation
AVP: Aortic valve repair
BAV: Bicuspid aortic valve
LVDd: Left ventricular diastolic diameter
LVDs: Left ventricular systolic diameter
LVEF: Left ventricular ejection fraction
